# Supplementary material for: Spontaneous Synchronization of Two Bistable Pyridine-Furan Nanosprings Connected by an Oligomeric Bridge
Source: Nanomaterials (Basel). 2023 Dec 19;14(1):3. doi: 10.3390/nano14010003 (PMC10780610; doi:10.3390/nano14010003)
Supplement: Supplementary file 1 [file nanomaterials-14-00003-s001.zip › nanomaterials-2744808-supplementary.pdf]

Supplementary Materials: Spontaneous synchronization of two bistable pyridine-furan nanosprings connected by an oligomeric bridge.

Anastasia A. Markina [1,2]*, Maria A. Frolkina[1,2], Alexander D. Muratov[1,2] and Vladislav S. Petrovskii [1,2], Alexander F. Valov[1,2], Vladik A. Avetisov [1,2]*

1. Simulation protocol

Morphology simulations were performed using the GROMACS simulation package. Details of the interaction parameters and experiment procedure are described elsewhere [17-19]. All calculations were performed in the NVT ensemble, in the simulation box of circa 52000 water molecules and size of 7nm x 7nm x 7nm using the canonical velocity-rescaling thermostat, as implemented in the GROMACS simulation package. The simulation was started from a random initial configuration and run to reach an equilibrated morphology. To study how the oligomers respond to a power load, the system simulation was continued for an additional 800 ns. The model error was estimated using the full width at 50% of the distribution curve maximum. To show that the results are independent of the specific bar length, different bar lengths were tested (see Figure S1).

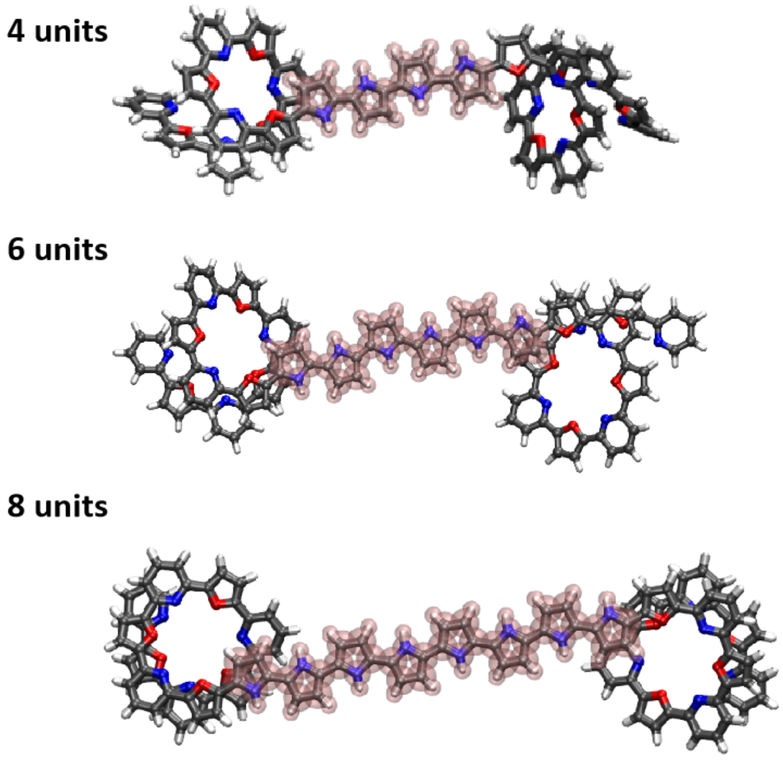

Figure S1. Two oligo-PF-5 springs connected with oligo-pyrrole bars of different lengths: 4, 6 and 8 units.

SV are observed for all three cases in a quite wide region (from 140pN to 220pN). The short connectivity bar (4 units) synchronizes the springs. However, the two states are not very well defined and overlapped on the histogram, due to steric blockings from the bar side (see Figure). In order to stretch springs are going up and at the same time rotate according to directions shown by red arrows. With a longer connectivity bar of length 8 relative movements of two springs are not blocked. In this case, two states are defined

better and do not overlap significantly on the histogram. The median length bar gives a better balance between the synchronization and splitting of two states.

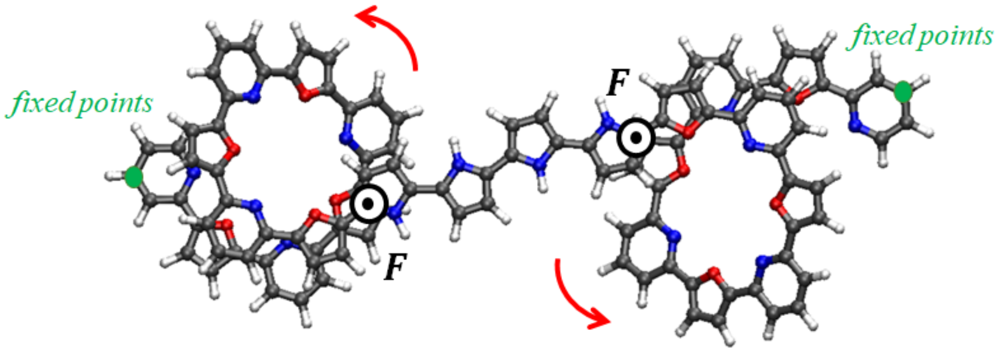

Figure S2. Two oligo-PF-5 springs connected with a short oligo-pyrrole bar of 4 units under the external load. Red arrows show the relative rotation of each spring.

2. Spontaneous vibrations and Stochastic Resonance

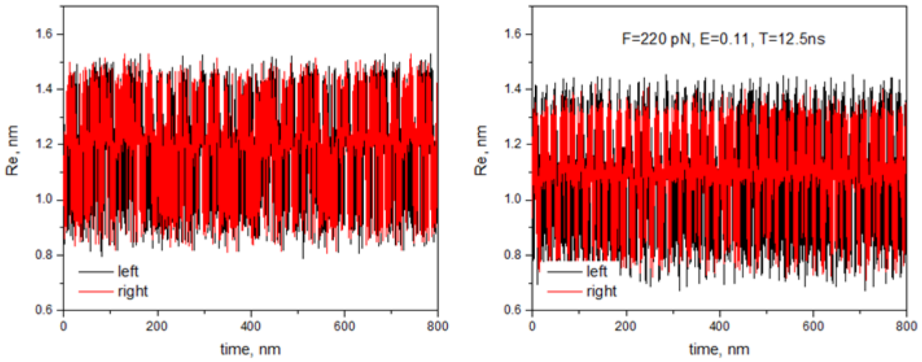

Figure S3. Example of trajectories of the end to end distance of two oligo-PF-5 springs connected with a long oligo-pyrrole bar of 6 units. (**left**) SV of the springs at F=220 pN; (**right**) SR of the springs induced by an oscillating field $E = E_0 \cos(2\pi \nu t) = E_0 \cos(2\pi t/T)$.

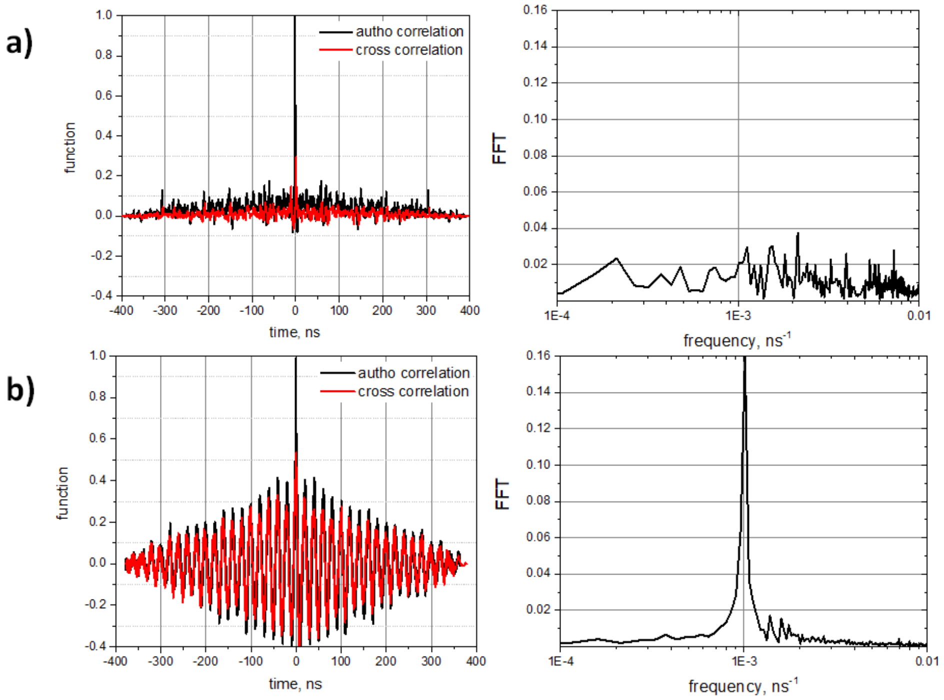

Figure S4. (**a**)) SV of the springs at $F \approx 220$ pN: correlation and autocorrelation functions of two springs (on the left) and Fourier spectrum of the autocorrelation function of the right spring (on the right). (**b**)) System with applied external field (SR): correlation and autocorrelation functions of two springs (on the left) and Fourier spectrum of the autocorrelation function of the right spring (on the right).
